# Supplementary material for: An acquired phosphatidylinositol 4-phosphate transport initiates T-cell deterioration and leukemogenesis
Source: Nat Commun. 2022 Jul 29;13:4390. doi: 10.1038/s41467-022-32104-7 (PMC9338045; doi:10.1038/s41467-022-32104-7)
Supplement: Supplementary file 1 — Supplementary Information [file 41467_2022_32104_MOESM1_ESM.pdf]

Supplementary Information

An acquired phosphatidylinositol 4-phosphate transport initiates T-cell deterioration and leukemogenesis

Wenbin Zhong[1,2], Weize Lin[1], Yingjie Yang[1], Dan Chen[1], Xiuye Cao[1], Mengyang Xu[1,3], Guoping Pan[1], Huanzhao Chen[1], Jie Zheng[1], Xiaoqin Feng[4], Li Hua Yang[5], Chaofeng Lai[1], Vesa M. Olkkonen[6], Jun Xu[3], Shuzhong Cui[2*], Daoguang Yan[1,2*]

[1]MOE Key Laboratory of Tumor Molecular Biology, Jinan University, Guangzhou, 510632, China.

[2]Affiliated Cancer Hospital and Institute of Guangzhou Medical University, Guangzhou, 510095, China.

[3]Research Center for Drug Discovery, School of Pharmaceutical Sciences, Sun Yat-Sen University, Guangzhou, 510006, China.

[4]Hematology and Oncology, Nanfang Hospital, Southern Medical University, Guangzhou, 510515, China.

[5]Pediatric Hematology Department, Zhujiang Hospital, Southern Medical University, Guangzhou, 510282, China.

[6]Minerva Foundation Institute for Medical Research, Biomedicum 2U, FI-00290 Helsinki, Finland. Department of Anatomy, Faculty of Medicine, FI-00014 University of Helsinki, Finland.

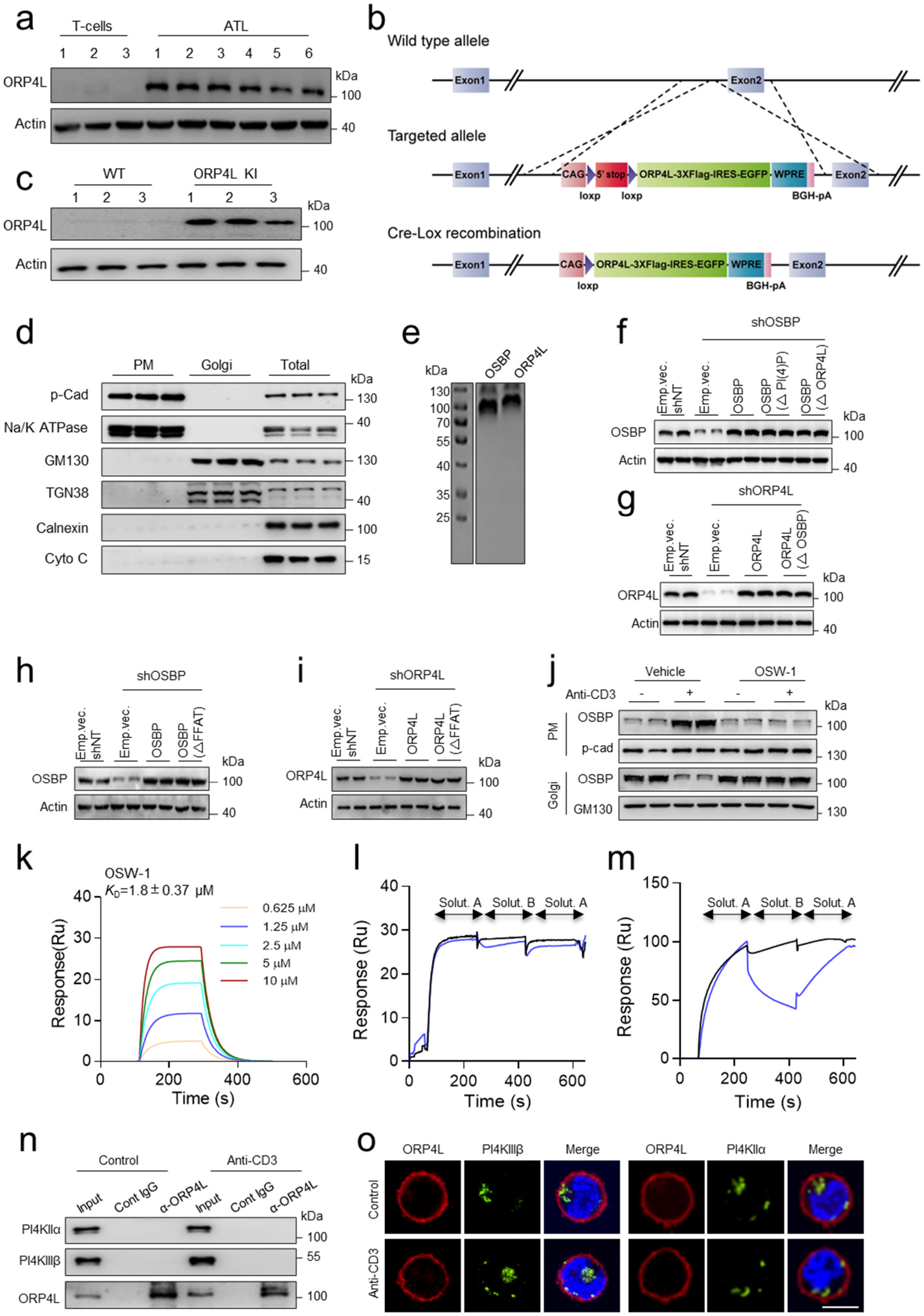
a
T-cells
ATL
1 2 3 1 2 3 4 5 6
ORP4L
Actin
kDa
100
40
b
Wild type allele
Exon1
Exon2
Targeted allele
CAG
5' stop
ORP4L-3XFlag-IRES-EGFP
WPRE
loxp
loxp
BGH-pA
Cre-Lox recombination
c
WT
ORP4L KI
d
PM
Golgi
Total
p-Cad
Na/K ATPase
GM130
TGN38
Calnexin
Cyto C
e
OSBP
ORP4L
f
shOSBP
Emp.vec. shNT
Emp.vec.
OSBP (△ PI(4)P)
OSBP (△ ORP4L)
g
shORP4L
ORP4L (△ OSBP)
h
OSBP (△FFAT)
i
ORP4L (△FFAT)
j
Vehicle
OSW-1
Anti-CD3
PM
Golgi
p-cad
k
OSW-1
K_D=1.8 ± 0.37 μM
0.625 μM
1.25 μM
2.5 μM
5 μM
10 μM
Response(Ru)
Time (s)
l
Solut. A
Solut. B
Response (Ru)
m
n
Control
Anti-CD3
Input
Cont IgG
α-ORP4L
PI4KIIα
PI4KIIIβ
o
ORP4L
PI4KIIIβ
Merge
PI4KIIα

Supplementary Figure 1. OSBP translocates from Golgi to plasma membrane in ORP4L KI T-cells. **a** Western blot analysis of ORP4L expression in normal human T-cells and ATL patient's T-cells. **b** Schematic representation of the constructs employed for generating T-cell-specific ORP4L overexpressing KI mice. **c** ORP4L expression in T-cells from 2-months old ORP4L KI and littermate wild-type mice. **d** Western blot analysis of membranes marker proteins from ORP4L KI T-cells. **e** SDS-PAGE gel showing pure recombinant OSBP and ORP4L proteins. **f, g** Western blot analysis of OSBP expression in ORP4L KI T-cells subjected to OSBP knockdown and re-expression with wild-type OSBP or [OSBP△PI(4)P] or [OSBP△ORP4L] (f), or subjected to ORP4L knockdown and re-expression with wild-type ORP4L or ORP4L(△OSBP) (g). **h, i** Western blot analysis of OSBP (h) and ORP4L (i) expression in ORP4L KI T-cells subjected to OSBP knockdown and re-expression of wild-type OSBP or OSBP(△VAPA) (h), or subjected to ORP4L knockdown and re-expression of wild-type ORP4L or ORP4L(△VAPA) (i). **j** Western blot analysis of OSBP levels in PM and Golgi of ORP4L KI T-cells upon OSW-1 treatment. **k** Kinetic analysis of OSW-1 binding to OSBP determined by SPR in real-time. Mean KD values ± SD (n = 3 biological replicates) are indicated. **l** Representative sensor grams of the ABA assay in the present (blue trace) or absence (black trace) of PI(4)P. **m** Representative sensor grams of the ABA assay in the presence (blue trace) or absence (black trace) of OSW-1. **n, o** Co-immunoprecipitation (n) and confocal microscopy (o) analysis the interaction of ORP4L with PI4KIIα and PI4KIIIβ in ORP4L KI T-cells with or without of anti-CD3 stimulation (10 μg/mL for 5 min at 37°C). Scale bar, 10 μm. Images of blot and confocal microscopy are representative of n=3 biological replicates with similar results. The same experiments were repeated twice in T-cells from two mice. Source data are provided as a Source Data file.

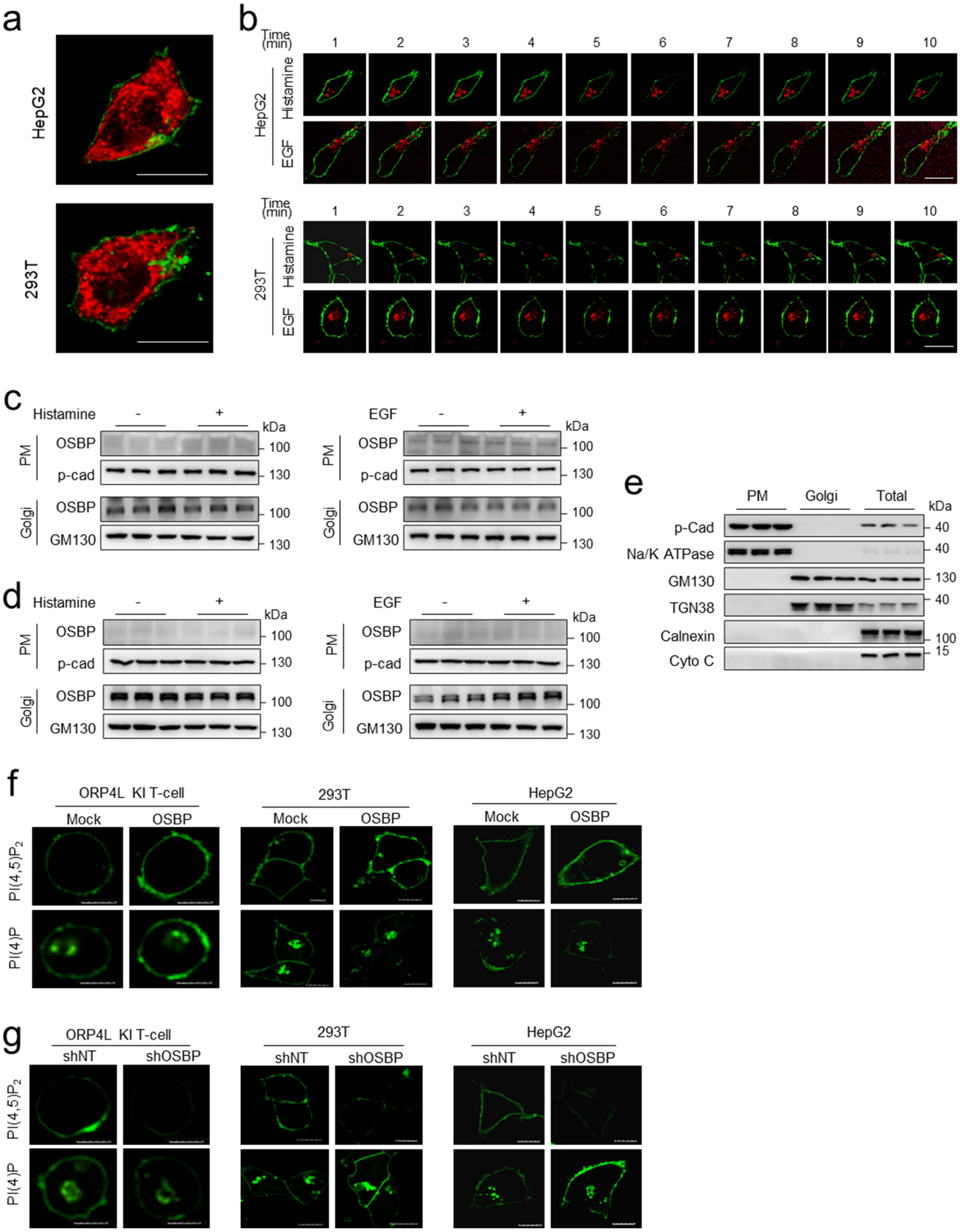
a
HepG2
293T
b
Time (min)
1
2
3
4
5
6
7
8
9
10
HepG2
Histamine
EGF
293T
c
Histamine
EGF
kDa
PM
Golgi
OSBP
p-cad
GM130
100
130
d
e
PM
Golgi
Total
p-Cad
Na/K ATPase
GM130
TGN38
Calnexin
Cyto C
40
15
f
ORP4L KI T-cell
Mock
OSBP
$PI(4,5)P_2$
PI(4)P
g
shNT
shOSBP

Supplementary Figure 2. The role of OSBP in regulation of the PI(4)P and PI(4,5)P_2 contents of different cell types. a Confocal microscopy analysis of localization of ORP4L in HegG2 and 293 T cells. The red color indicates ORP4L, the green color indicates the PM marker p-Cad. Scale bar, 10 μm. **b** Time course of DsRed-OSBP protein, the PI(4,5)P_2 probe GFP-$PH_{PLC\delta1}$ at the PM of HepG2 cells (upper) and 293T cells (lower). After baseline measurement, cells were treated with histamine (10 μg/mL at 37°C) or EGF (50 ng/mL at 37°C) and the changes in fluorescence intensity were monitored by time-lapse microscopy. Scale bar, 10 μm. **c, d** Western blot analysis of OSBP protein levels in the PM and Golgi of of HepG2 (c) and 293T cells (d). Cells were treated with histamine (10 μg/mL at 37°C) or EGF (50 ng/mL at 37°C) for 5 min before PM and Golgi isolation. **e** Western blot analysis of membranes marker proteins from HepG2 cells. **f** Fluorescence images of the PI(4,5)P_2 probe GFP-$PH_{PLC\delta1H}$ or PI(4)P probe GFP-$P4M_{SidM}$ in ORP4L KI T-cells, 293 T or HepG2 cells upon OSBP overexpression. Scar bar, 10 μm. **g** Fluorescence images of the PI(4,5)P_2 probe GFP-$PH_{PLC\delta1}$ or PI(4)P probe GFP-$P4M_{SidM}$ in ORP4L KI T-cells, 293 T or HepG2 cell upon OSBP knockdown. Scar bar, 10 μm. Images of blot and confocal microscopy are representative of n=3 biological replicates with similar results. The same experiments were repeated twice. Source data are provided as a Source Data file.

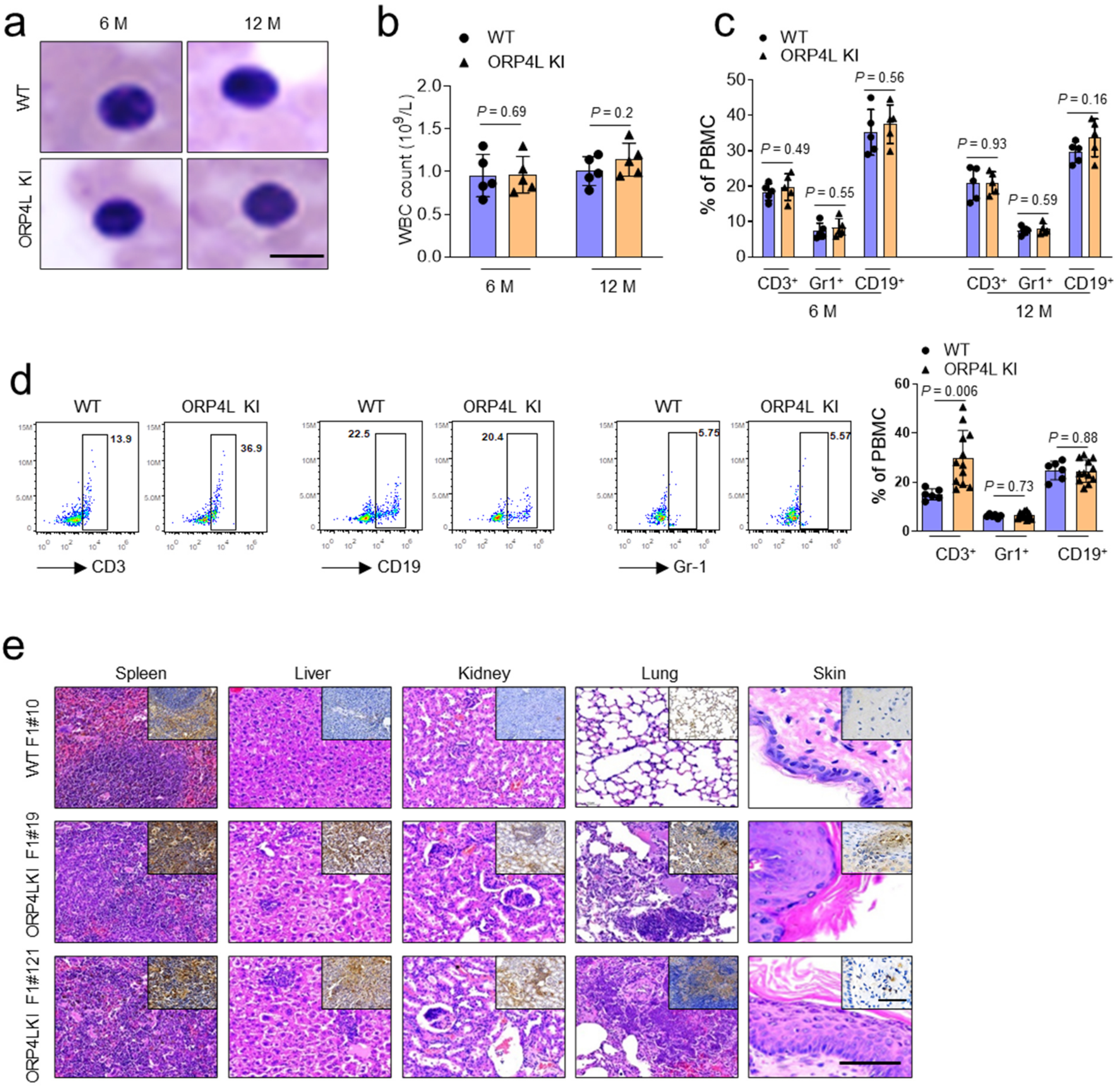

Supplementary Figure 3. Flow cytometry and pathological findings reveal a T-cell leukemia phenotype in ORP4L KI mice. a Representative images of peripheral blood smears from ORP4L KI mice and littermate controls at 6 and 12 months of age. Scale bar, 10 μm. **b** White blood cell count of ORP4L KI and littermate wild-type mice at 6 and 12 months of age. Black triangles present in dividual data points for n=5 mice. **c** Percentage of $CD3^+$ T-cells, $CD19^+$ B cells and Gr-1^+ granulocytes in ORP4L KI mice and littermate wild-type mice at 6 and 12 months of age. Black triangles present in dividual data points for n=5 mice. **d** Flow cytometry

analysis of cell-surface markers CD3, CD19 and Gr-1 in lymphomatous cells. The right panel illustrates the percentage of indicated cells analyzed from ORP4L KI mice at moribund state and littermate wild-type mice at the same age. Black triangles present in dividual data points for n=12 (ORP4L KI) and n=6 (WT) mice. **e** Representative H&E-staining showed T-cells infiltration in spleen, liver, kidney, lung, and skin in ORP4L KI mice (F1#19, F#21) at moribund state. Wild-type littermate control (F1#10) at the same age is shown. Scale bars, 100 μm. Images of (a, e) are representative of n=3 biological replicates with similar results. In panel (b-d), data are presented as Mean ± SD, two-tailed unpaired *t*-test with a confidence interval of 95%. Source data are provided as a Source Data file.

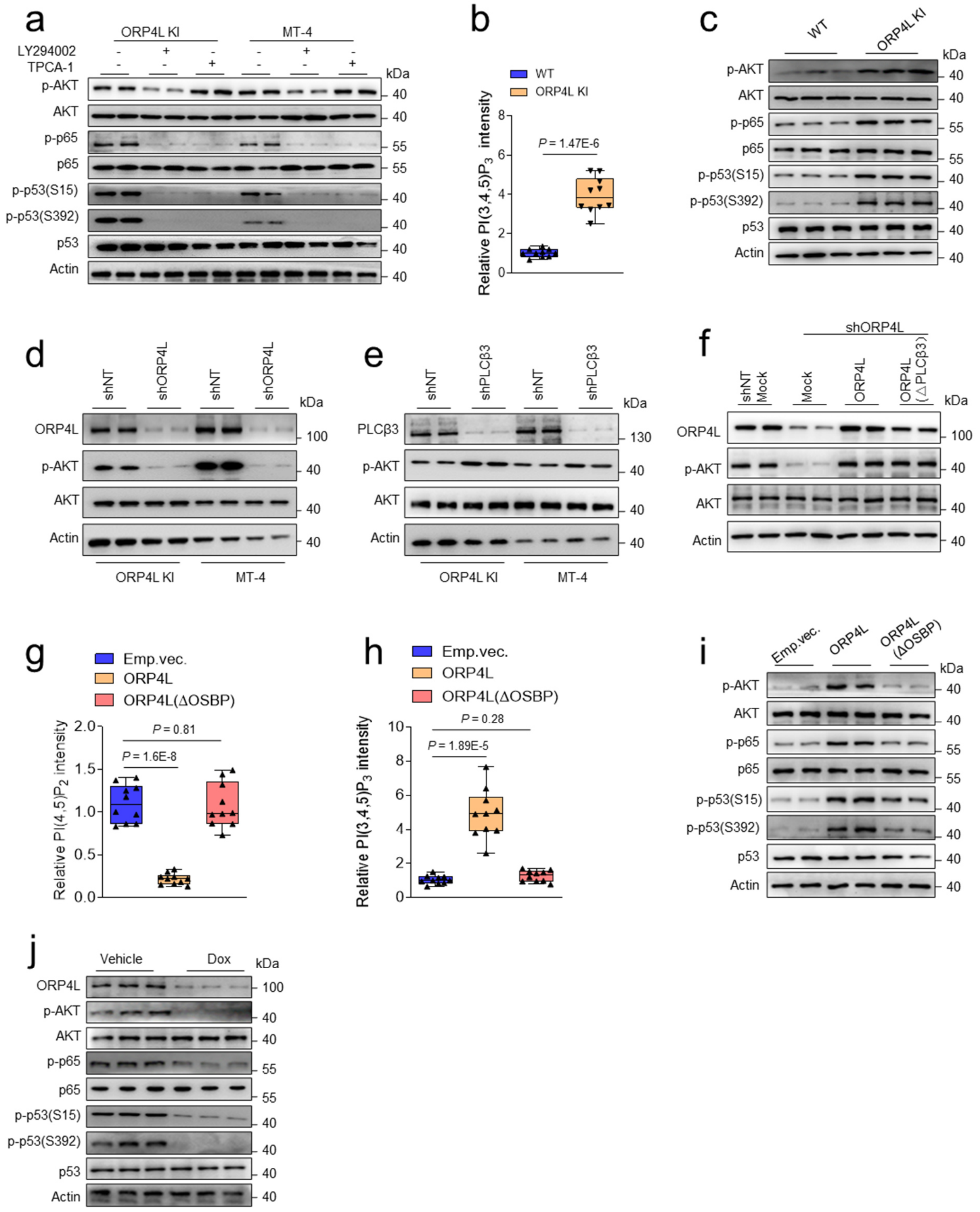

Supplementary Figure 4. PI(3,4,5)P3 generation and downstream pathway analysis in pre-malignant T-cells. a AKT activation, p65 and p53 phosphorylation in T-cells from one of

ORP4L KI mouse and MT-4 cell line upon AKT inhibitor (LY294002, 10 μM for 24 hr) or NF-κB inhibitor (TPCA-1, 5 μM for 24 hr) treatments. **b** PI(3,4,5)P_3 contents in T-cells of wild-type and ORP4L KI mice at the age of 6-months. Black triangles present in dividual data points for n=10 cells from one mouse. **c** AKT activation, p65 and p53 phosphorylation in T-cells from one of wild-type and ORP4L KI mice at the age of 6-months. **d, e** AKT activation in T-cells from one of ORP4K KI mouse, and MT-4 cells upon ORP4L (d) or PLCβ3 (e) knockdown. **f** AKT activation in T-cells from one of ORP4L KI mouse subjected to ORP4L knockdown and re-expression with wild-type ORP4L or ORP4L with PLCβ3 binding site mutations. **g, h** PI(4,5)P_2 and PI(3,4,5)P_3 contents in T-cells transduced with lentivirus carrying ORP4L or ORP4L with OSBP binding sites mutations. The T-cells were transduced and cultured *in vitro* for 2 weeks before analysis. Black triangles present in dividual data points for n=10 cells from one mouse. **i** AKT activation, p65 and p53 phosphorylation in T-cells treated as panel (g). **j** Western blot analysis of ORP4L knockout and downstream signaling pathway in T-cells from one of ORP4L KI mouse transduced with constitutively expressed Cas9 and Dox-inducible ORP4L sgRNA lentiviral vectors with or without treatment with Dox (1 mg/ml) for 72 hr *in vitro*. Images of blot are representative of n=3 biological replicates with similar results. The same experiments were repeated twice in T-cells from two mice. In each box plot, the central mark indicates the median, the bottom and top edges of the box indicate the interquartile range, and the whiskers represent the maximum and minimum point. Two-tailed unpaired *t*-test (b) and One-way ANOVA test (g, h) with a confidence interval of 95% was used to compute statistics. *P* values are indicated in the figures. Source data are provided as a Source Data file.

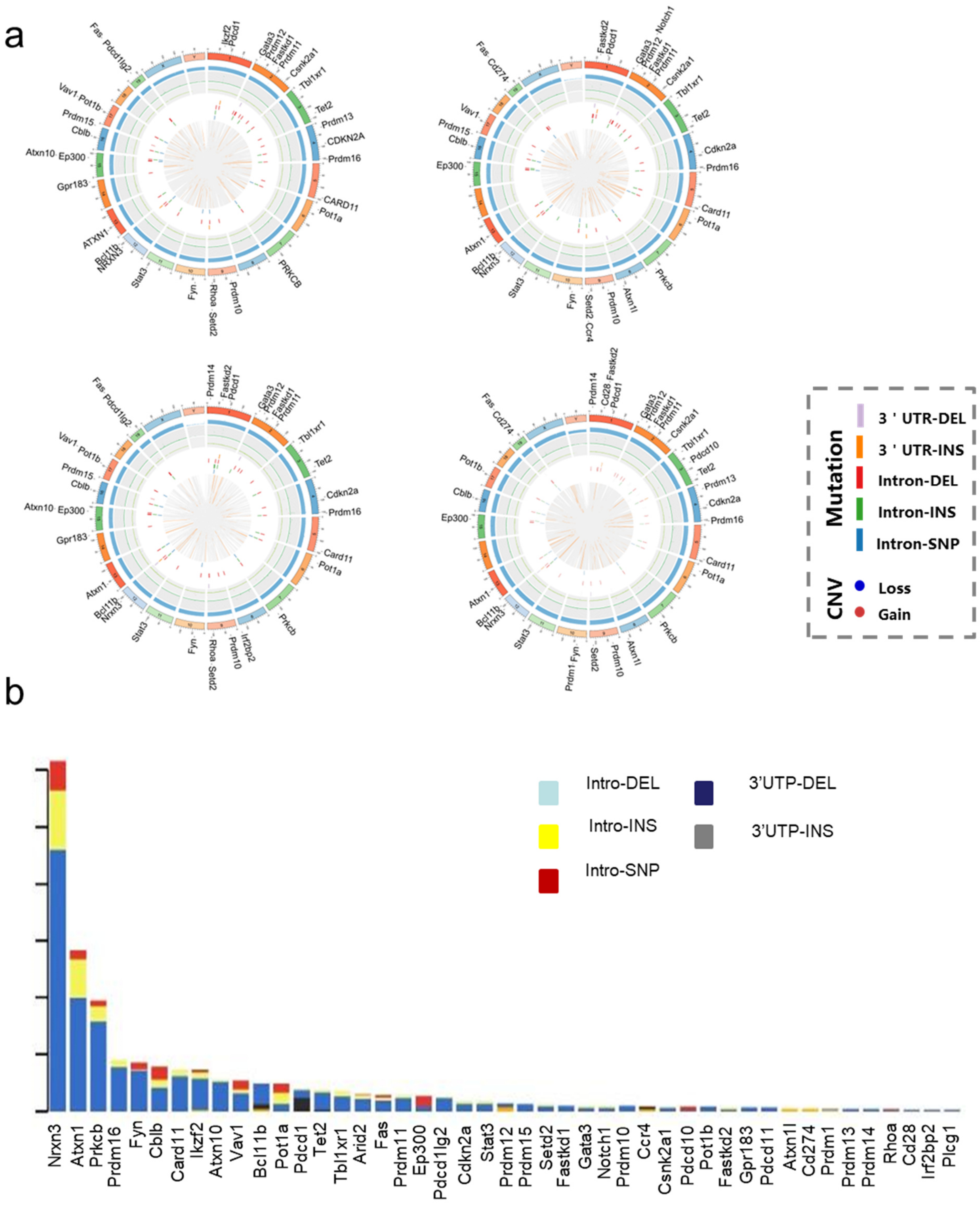

Supplementary Figure 5. Overview of the genome of ORP4L KI T-cells. a Circus plot depicting the somatic alterations for T-cells in ORP4L KI mice. Somatic mutations, copy number variations (CNVs) and structural variations (SVs) are shown along chromosome ideograms. **b**

Frequencies and types of somatic mutations identified by targeted capture sequencing in T-cells from ORP4L KI mice (n=4 mice). Significant somatic mutations and focal CNVs were referenced to the landscape of somatic alterations in human ATL.

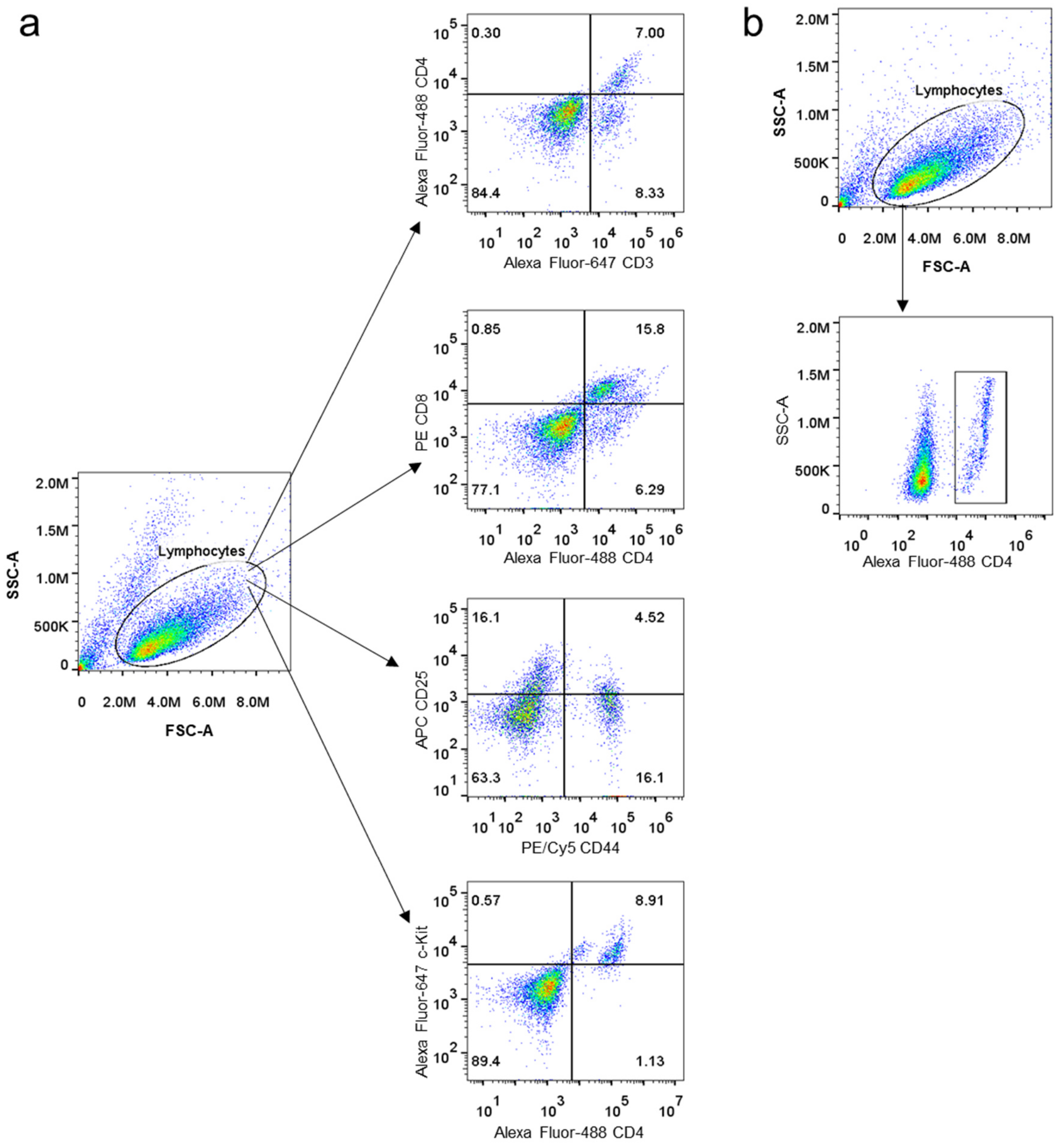

Supplementary Figure 6. Gating strategy for flow cytometer analysis. a Used in Figure 4c and Figure 6f. **b** Used in Figure 4d and 4e.

Supplementary Table 1. Details of the clinical samples used.

Sample	Diagnosis	WBC (/ul)	sIL2R (U/ml)	Proviral load (%)
1	Acute	54100	55200	201.4
2	Acute	8900	6100	112.6
3	Acute	29800	95000	68.9
4	Smoldering	6000	11820	58.7
5	Chronic	9700	8970	113.5
6	Acute	21600	5530	84

Supplementary Table 2. Oligonucleotide primers used for cDNA constructs.

Constructs	Forward primer 5’- 3’	Reverse primer 5’- 3’
ORP4L-pcDNA4 HisMaxC / pGEX4T-1	ATTtctaga[1]ATGGGGAAAGCGGCGGT	ATTtctagaGTGGCGCTCAGAAGATGTTGGGGCACATATGCCA
OSBP-pcDNA4 HisMaxC / pGEX4T-1	ATTggatccATGGCGGCGACGGAGCTGAGAGGAG	ATTgtcgacTCAGAAAATGTCCGGGCATGAGCTC
OSBP-DsRed-C1	TCGAGCTCAAGCTTCgaattcTATGGCGGCGACGGAGCTGAGAGG	TTATCTAGATCCGGTggatccTCAGAAAATGTCCGGGCATGAGCT
OSBP(△ORP4L)-pcDNA4 HisMaxC	Forward 1:<br>ATTggatccATGGCGGCGACGGAGCTGAGAGGAG<br>Forward 2:<br>AGATCAAACAGGTCAACGAAAAATGGCAAAAGTCACTACAGTATG	Reverse 1:<br>ATTgtcgacTCAGAAAATGTCCGGGCATGAGCTC<br>Reverse 2:<br>TGTAGTGACTTTTGCCATTTTTCGTTGACCTGTTTGATCTTTTCA
OSBP(△PI(4)P)-pcDNA4 HisMaxC	Forward:<br>ATTggatccATGGCGGCGACGGAGCTGAGAGGAG<br>Forward FF/AA:<br>TGAAGCGGCGGATGCACCTGAGATCATCACCA<br>Forward HH/AA:<br>AGTGCGGCGCCCCCTGCTGCTGCGCACCATGCTGAGTCC<br>Forward K/A:<br>ATGCGGAGGCGCAGCGCCTGGAGGAAAAACAAAGACTT	Reverse:<br>ATTgtcgacTCAGAAAATGTCCGGGCATGAGCTC<br>Reverse FF/AA:<br>CAGGTGCATCCGCCGCTTCATTCTCATCATCT<br>Reverse HH/AA:<br>GCAGCAGGGGGCGCCGCACTCACCTGTTCACAGAGGGAT<br>Reverse K/A:<br>CAGGCGCTGCGCCTCCGCATTTGCTTCATCCCAGC

ORP4L (△FFAT)-pcDNA4 HisMaxC	Forward △FFAT: GAAGATGAAGATACCGACTCCACATCCTTCAT	Forward △FFAT: GAAGGATGTGGAGTCGGTATCTTCATCTTCCTC
OSBP (△FFAT)-pcDNA4 HisMaxC	Forward △FFAT: GAAGATGATGAGAATATCATCACCATGCCTGAA	Forward △FFAT: AGGCATGGTGATGATATTCTCATCATCTTCAT

[1] Restriction sites are indicated in lower case letters
[2]For human species.
[3]For mouse species.

Supplementary Table 3. The targeted shRNA and sgRNA sequences used.

Genes	Sequences
shNT	GCATTGGTCGTCTCTATTA
shORP4L	TCAGAGTCAAGCTCAGGTGTA
shOSBP	AGGCTACCAGCGGCGATGG
sgORP4L	CACTGGTCAGCAAGATACCACAAGAGT
shPI4K IIIα	GCGTCTCATCACATGGTACAA
shPIP5KB	ACGACAGGCCTACACTCTATT
shVAPA	GGTAGCACATTCGGATAAACCTGGA
shPLCβ3-mouse	TCAAGAACATTCTGAAGAT
shPLCβ3-human	AGATGAGGGACAAGCATAAGAAGGA
shPI4KIIα	CGUUCUCUCAGGAGAUCAAAG
