# Supplementary material for: An acquired phosphatidylinositol 4-phosphate transport initiates T-cell deterioration and leukemogenesis
Source: Nat Commun. 2022 Jul 29;13:4390. doi: 10.1038/s41467-022-32104-7 (PMC9338045; doi:10.1038/s41467-022-32104-7)
Supplement: Supplementary file 3 — Description of Additional Supplementary Files [file 41467_2022_32104_MOESM3_ESM.pdf]

Description of Additional Supplementary Files

File Name: Supplementary Movie 1
Description: Time course of the PI(4,5)P_2 probe GFP-$PH_{PLC\delta 1}$ at the PM of ORP4L KI T-cells subjected to PI4K IIIα or PIP5KB knockdown.

File Name: Supplementary Movie 2
Description: Time course of the PI(4)P probe GFP-$P4M_{SidM}$ at PM and Golgi complex of ORP4L KI T-cells subjected to PI4K IIIα knockdown.

File Name: Supplementary Movie 3
Description: Time course of the PI(4)P probe GFP-$P4M_{SidM}$ at PM and Golgi complex of ORP4L KI T-cells.

File Name: Supplementary Movie 4
Description: Time course of the DsRed-OSBP protein at the PM and Golgi of ORP4L KI T-cells.

File Name: Supplementary Movie 5
Description: Time course of the cholesterol probe mCherry-D4H at the PM and Golgi of ORP4L KI T-cells.

File Name: Supplementary Data 1
Description: Lipidomics mass spectrometry data of PM isolated from wild-type and ORP4L KI mice (n=3 biologically independent mice). WT, wild-type. QC, quality control.
